# Cortistatin-14 Exerts Neuroprotective Effect Against Microglial Activation, Blood-brain Barrier Disruption, and Cognitive Impairment in Sepsis-associated Encephalopathy

**DOI:** 10.1155/2022/3334145

**Published:** 2022-09-13

**Authors:** Qiang Wen, Qian Ding, Jinchao Wang, Yanhua Yin, Shangchen Xu, Yuanrong Ju, Hongsheng Ji, Bin Liu

**Affiliations:** ^1^Department of Oncology, Shandong Provincial Hospital Affiliated to Shandong First Medical University, Shandong First Medical University, Jinan 250021, China; ^2^Department of Gastroenterology, Shandong Provincial Hospital Affiliated to Shandong First Medical University, Shandong First Medical University, Jinan 250021, China; ^3^Department of Neurosurgery, Shandong Provincial Hospital Affiliated to Shandong First Medical University, Shandong First Medical University, Jinan 250021, China; ^4^Department of Gastroenterology, Taian Hospital of Traditional Chinese Medicine, Tai'an 271000, China; ^5^Department of Critical Care Medicine, Shandong Provincial Hospital Affiliated to Shandong First Medical University, Shandong First Medical University, Jinan 250021, China; ^6^School of Medicine, Shandong University, Jinan 250012, China; ^7^Shandong University of Traditional Chinese Medicine, Jinan 250011, China

## Abstract

Sepsis-associated encephalopathy (SAE) is a life-threatening deterioration of mental status in relation to long-term and disabling cognitive dysfunction that is common in intensive care units worldwide. Cortistatin-14 is a neuropeptide structurally resembling somastostatin, which has been proven to play a crucial role in sepsis. The present study aimed to explore the neuroprotective role of cortistatin-14 in sepsis-associated encephalopathy and its underlying mechanisms in a mouse model. A septic mice model was established using the cecal ligation and puncture (CLP) method. The novel object recognition test (NORT), open field test (OFT), elevated plus maze test (EPMT), and tail suspension test (TST) were used to explore the behavioral performance of the mice. Transmission electron microscopy was used to observe the microstructure of the blood-brain barrier (BBB). Evans Blue staining was used to examine the integrity of the BBB. Immunofluorescence was used to examine the morphology and infiltration of microglia. A multiplex cytokine bead array assay was used to determine cytokine and chemokine levels in mouse serum and brain tissues. NORT revealed that cortistatin treatment improved cognitive impairment in septic mice. OFT, EPMT, and TST indicated that cortistatin-14 relieved the anxiety-related behaviors of CLP mice. In addition, cortistatin-14 treatment decreased the levels of various inflammatory cytokines, including interleukin-1*β*, interleukin-6, interferon-*γ*, and tumor necrosis factor-*α* in both the serum and brain of septic mice. Cortistatin reduced sepsis-induced blood-brain barrier disruption and inhibited microglial activation after the onset of sepsis. Cortistatin exerts neuroprotective effects against SAE and cognitive dysfunction in a CLP-induced mouse model of sepsis.

## 1. Introduction

Sepsis-associated encephalopathy (SAE) is a diffuse brain dysfunction secondary to severe sepsis in the absence of a direct central nervous system (CNS) infection, characterized by alteration of consciousness. SAE is frequently encountered in up to 70% of critically ill patients with severe systemic infections. Additionally, the severity of SAE is associated with increased mortality [1]. Thus, exploring the mechanism underlying brain injury in the setting of systemic sepsis will aid in developing strategies to treat SAE.

The key mechanisms associated with SAE were generally considered to include excessive microglial activation, endothelial barrier dysfunction, and blood-brain barrier (BBB) disruption [2]. The BBB plays a critical role in maintaining the homeostasis of the brain microenvironment and restricting the infiltration of inflammatory cells from the peripheral blood into the CNS [2]. Increasing evidence suggests that BBB dysfunction is closely related to the pathophysiology of sepsis and SAE, leaving the CNS highly vulnerable to neurotoxic factors, such as free radicals, inflammatory cytokines, intravascular proteins, and circulating leukocytes [3]. Microglial activation is a major resident immune cell type in the CNS. In sepsis, sustained inflammation leads to persistent microglial activation and increased BBB permeability [4]. While it is intended to be a defensive immune response against sepsis, microglial activation generates a cytotoxic environment that induces the release of reactive oxygen species, nitric oxide, and glutamate [5]. These observations strongly suggest that reactive microglia likely contribute to the exacerbation of SAE pathology and BBB dysfunction [6].

The cyclic neuropeptide related to somatostatin, cortistatin, has been proved to act as a “macrophage-deactivating” agent [7]. Despite its similar molecular structure, cortistatin is significantly more efficient than somatostatin in protecting against sepsis, inflammatory bowel disease, and collagen-induced arthritis [7]. Cortistatin treatment protects against septic shock-associated histopathology in various target organs, including the liver, lung, and intestine [8]. Also, cortistatin has been reported to exert a neuroprotective effect in various CNS diseases, including autoimmune encephalomyelitis, neurodegenerative diseases, and bacterial meningoencephalitis [9–11]. However, little is known about the neuroprotective role of cortistatin-14 in SAE.

Based on these reports, the present study evaluated the role of cortistatin-14 in neural and cognitive protection via the meditation of microglial activation and BBB dysfunction in the setting of sepsis using a mouse model.

## 2. Materials and Methods

### 2.1. Microarray-based profiling

The SAE-related murine brain mRNA dataset GSE167610, which contains eight control and eight disease samples, was downloaded from the GEO database. The sequencing platform annotation file GPL6885 was used for gene ID conversion. R “limma” package was used for differential analysis to identify the differentially expressed mRNAs with |log_2_FC| >1 and *p-*value <0.05 as the threshold.

### 2.2. Multiplex cytokine bead array assay

Cytokine and chemokine levels were measured in mouse serum (20× diluted) and brain samples (10× diluted) using the MILLIPLEX MAP Mouse Cytokine/Chemokine Magnetic Bead Panel (MCYTMAG-70 K-PX32; Millipore), following the manufacturer's instructions.

### 2.3. Animals

Male 8–12-week-old wild-type C57BL/6 J mice were purchased from the Beijing Vital River Laboratory Animal Technology Co., Ltd. (Beijing, China). The mice were housed in a specific pathogen-free (SPF) facility at the Shandong Provincial Hospital Affiliated to Shandong First Medical University for at least one week before inclusion in the experiments.

### 2.4. Development of mouse cecal ligation and puncture (CLP) model and treatment protocol

A mouse model of sepsis was induced using the CLP method, as previously described [12]. Briefly, mice were anesthetized by intraoperitoneal (i.p.) administration of 1.5% isoflurane, and the head and limbs were fixed. The cecum was exposed by a small midline in the lower abdomen, ligated using 3.0 silk and punctured using a 21-gauge needle. A small amount of feces was then extruded using forceps. The cecum was relocated into the abdominal cavity and the abdomen was closed using sutures. Sham-operated mice were subjected to the same operation procedure but without CLP.

Animal experiments were approved by the Animal Ethics Committee of Shandong Provincial Hospital Affiliated to Shandong First Medical University and performed in strict accordance with the Guide for the Care and Use of Laboratory Animals published by the US National Institutes of Health.

Mice were randomly assigned to three groups: sham (sham-treated with corn oil), CLP (CLP treated with corn oil), and cortistatin groups (CLP treated with cortistatin-14, GlpBio, Montclair, CA). The reagents were administered intraperitoneally 30 min after the operation. The mice were euthanized at 7d after the operation.

### 2.5. Open-Field Test (OFT)

The OFT was used to measure the exploratory activity and anxiety-like behavior of the mice. Each mouse was placed at the center of an open-field device (50 cm ×50 cm ×50 cm). The central zone was defined as a square that was 10 cm from the wall. The time spent exploring the central zone was recorded for 5 min and quantified using the SMART system (Panlab Harvard Apparatus; Barcelona, Spain). The central duration was calculated using the following formula: central duration (%) = (time spent exploring the central zone/300 s) ×100%.

### 2.6. Tail-suspension test (TST)

TST was conducted according to previous studies with some modifications [13, 14]. The tail of each mouse was suspended at the height of 50 cm using adhesive tape. The duration of immobility over the 5 min test period was recorded and quantified using the SMART system (Panlab Harvard Apparatus) [15].

### 2.7. Elevated plus maze test (EPMT)

Each mouse was placed in the center zone (10 cm × 10 cm) of the elevated plus maze, facing one of the open arms (40 cm above the floor). The number of entries and time spent in both open arms (30 cm × 5 cm) and enclosed arms (30 cm × 5 cm, with 15 cm-high walls) were recorded using the SMART system (Panlab Harvard Apparatus) for 5 min [15]. The percentages of open arm entries and time spent in the open arms were calculated as the number of open arm entries divided by the total number of arm entries and the time spent in the open arms divided by the total time, respectively [13].

### 2.8. Novel object recognition test (NORT)

NORT was used to investigate the working and memory of rodents. The procedure was performed according to a previously reported protocol with some modifications [16]. The experimental procedure consisted of three sessions: habituation, training, and testing. Each mouse was placed in a Plexiglas cage (50 cm ×50 cm ×50 cm) with an exchangeable floor. Habituation was carried out for 15 min on the first three consecutive days when the mice were positioned into empty chambers without any object. Twenty-four hours after habituation, mice entered the training session. In this session, the mice were placed in the same chamber facing the wall opposite to two identical objects (A1 and A2) and allowed to explore the objects for 5 min. The time spent exploring each object was recorded. After a delay of 3 h (short-term memory), the mouse was returned to the chamber with one of the familiar objects (A2) replaced by a novel object (A3). In the test session, mice were allowed to freely explore the familiar object (A1) and the new object (A3) for 5 min. The exploration time for each object during the test session was recorded using a SMART system (Panlab Harvard Apparatus). The discrimination ratio was calculated as follows: time of exploration of the novel object/(time of exploration of the familiar object + time of exploration of the novel object) ×100. An index greater than 0.5 indicates a preference for the novel object. The apparatus and the objects were thoroughly cleaned with 75% ethanol between each trial to control olfactory cues.

### 2.9. Transmission electron microscopy

The 1 mm^3^ fragments of prefrontal cortex tissue were fixed by immersing in 3% glutaraldehyde in 0.2 M phosphate buffer (0.874 g NaH_2_PO_4_, 5.158 g Na_2_HPO_4_ in 100 mL distilled water) for more than 24 h, post-fixed with osmic acid for 2 h and processed for transmission electron microscopy. The ultrathin sections were stained with uranyl acetate and lead citrate. Finally, the ultrastructure of the endothelial cells, neuronal mitochondria and synapses was examined under a transmission electron microscope (JEOL-1011; JEOL, Tokyo, Japan).

### 2.10. Evans blue staining

The integrity of the mouse BBB was evaluated using Evans blue staining. The leakage of Evans blue dye in the brain tissues of five mice in each group was analyzed one day after surgery. Three hours before euthanasia, the mice received an intravenous injection of 2% Evans blue dye (E2129, EBD, Sigma-Aldrich, St. Louis, MO; 6 mL/kg saline). The concentration of Evans Blue in the brain was quantified at 610 nm using spectrophotometry, and a quantification plot was plotted.

### 2.11. Immunofluorescence

Mouse brain sections were preincubated with 0.3% Triton X-100 (HFH10, Thermo Fisher Scientific) in PBS for 10 min and blocked with 1% BSA (37525, Thermo Fisher Scientific) and 0.1% Triton X-100 for 1 h. The sections were then immunostained overnight at 4°C with primary antibodies against mouse Iba1 (ab178846, rabbit, monoclonal, 1 : 500, Abcam, Cambridge, UK). The sections were then incubated with AF488-conjugated secondary antibody for 1 h at 25°C. Images were acquired using a confocal microscope (Leica Microsystems GmbH, Wetzlar, Germany) in five different fields selected from each section. Fluorescent cells were counted using the ImageJ software.

### 2.12. Statistical analysis

The measurement data were described as mean ± standard deviation, and SPSS 21.0 software and GraphPad Prism 5.0 were used to analyze the data. Statistical significance was measured using the paired t-test (two-group paired data), unpaired t-test (two-group unpaired data), one-way ANOVA (multi-group data), or repeated measures ANOVA (multi-group data). The Kaplan-Meier method was used to assess survival, and the results were compared between groups with a log-rank test. Differences were considered statistically significant at *P* < 0.05.

## 3. Results

### 3.1. Cortistatin is decreased in SAE mice

**Figure 1:**
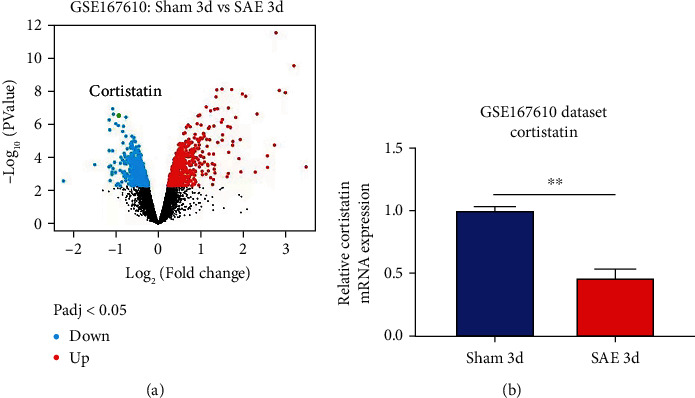
Analysis of the SAE-related murine brain mRNA dataset GSE167610. (a) Volcano plot of differentially expressed RNA between (|FC| >1, *P* < 0.05). (b) Decreased cortistatin mRNA expression in the SAE mouse brain compared to sham mouse brain.

Analysis of the GSE167610 dataset suggested that 1313 mRNA were expressed in various ways (Figure 1(a)). Among these, decreased expression of cortistatin mRNA in the brains of SAE mice compared to sham mice in the acute phase of sepsis was identified (three days after peritoneal contamination and infection [PCI]; fold change = 0.482, *t* = 11.92, *P* = 2.33 × 10^−7^, adjusted *P* = 1.67 × 10^−4^) (Figure 1(b)).

### 3.2. Cortistatin-14 improved the survival and behavioral performance of CLP mice

**Figure 2:**
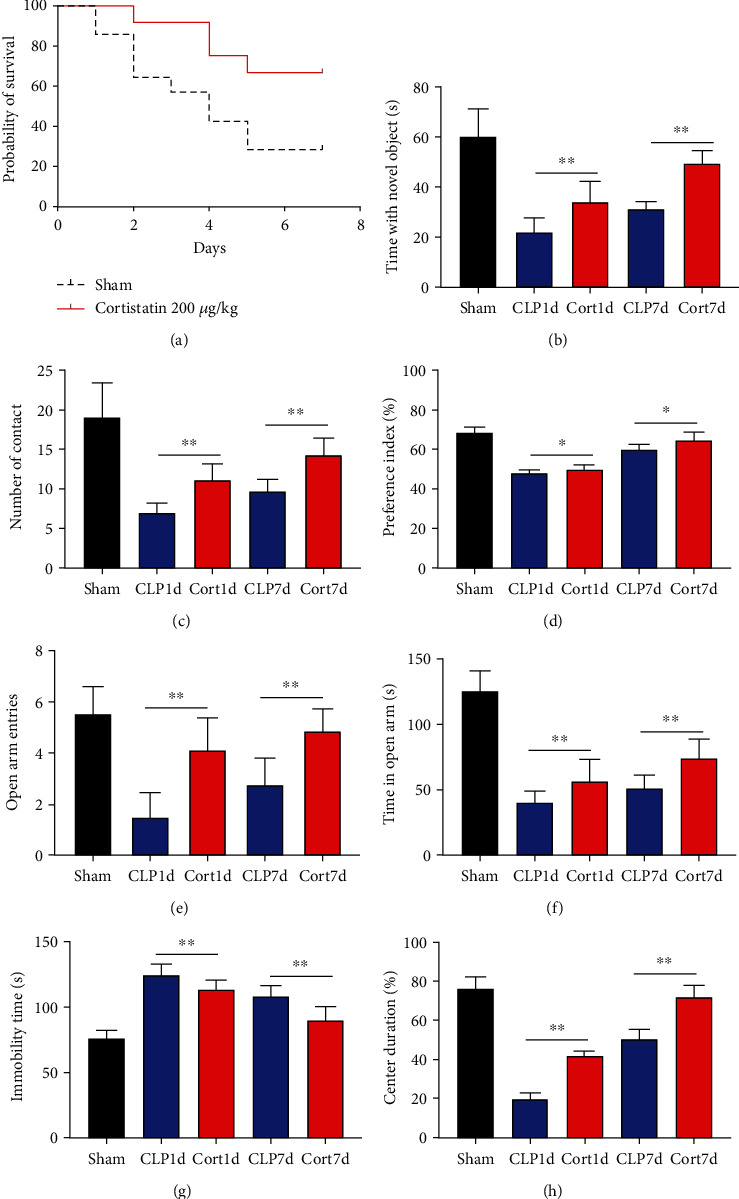
Cortistatin improved overall survival and cognitive and emotional impairement of CLP-induced sepsis in mice. (a) Survival curve. Four of 14 mice of the CLP group survived on day 7. Eight of 12 mice of the cortistatin group survived. (b)–(d) Time spent with a novel object, contacts with the novel object, and preference index in the novel object recognition test, respectively. (e),and (f) Entries of the open arms and time consumed in the open arms in the elevated plus maze test, respectively. (g) Total immobility time during 5 min in the tail suspension test. (h) Percentage of center duration in the 5 min in the open field test. Data were shown as mean ± standard deviation (n = 8 per group). ∗*P* < 0.05 and ∗∗*P* < 0.01.

To investigate the potential therapeutic effect of cortistatin-14 in SAE, we treated a CLP septic murine model by intraperitoneal administration of cortistatin at various doses (100–500 *μ*g/kg). The results indicated that cortistatin exerted a protective effect on the total survival of CLP mice (Figure 2(a)), and the effect was dose-dependent (Figure S1).

We then examined the therapeutic effect of cortistatin-14 (500 *μ*g/kg) on behavioral performance in post-septic mice. The tests were performed one day and seven days after CLP operation.

NORT was used to investigate the hippocampal mediated learning and memory in all the surviving mice. During the training session, all groups showed similar exploration times for two identical objects (data not shown). However, during the test session, CLP mice showed decreased exploratory preference and spent less time exploring the novel object both at days 1 and 7 after CLP surgery when compared to the sham mice. In contrast, cortistatin-treated CLP mice displayed improved recognition memory compared with the CLP group (Figures 2(b)–2(d). Consequently, we performed EPMT, OFT and TST to investigate the emotional behaviors of each group one day and seven days after the CLP surgery. In the EPMT, the number of entries and the amount of time spent in the open arm were comparable between the groups (Figures 2(e) and 2(f). Additionally, the TST, an anxiety-related behavioral test, revealed that the mean immobility time was significantly shorter in the cortistatin-treated mice than in septic mice (Figure 2(g)). Similarly, the OFT was used to examine the locomotor activitiy and anxiety-like behaviors. CLP mice spent lesser time in the central zone of the field than that of the sham mice, but spent more time than the cortistatin-treated mice (Figure 2(h)).

In general, these results indicate that the onset of sepsis significantly impaired the the cognitive function and emotion of the mice, while cortistatin-14 treatment prevented these adverse events.

### 3.3. Cortistatin treatment mitigates local and systemic inflammatory responses and inhibits microglia activation in septic mice

**Figure 3:**
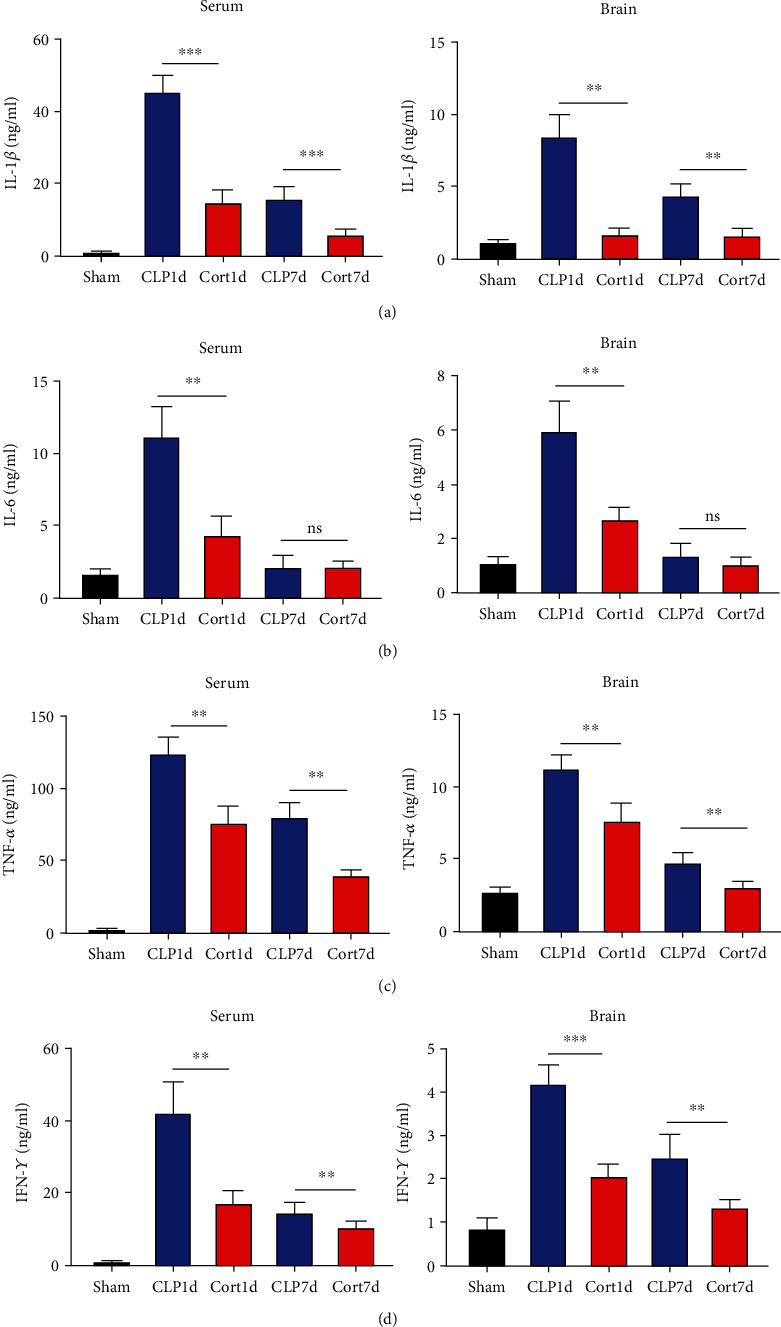
Cortistatin treatment decreased systemic (serum, left panels) and regional (brain, right panels) inflammatory cytokines levels. Levels of IL-1 *β* (a), IL-6 (b), TNF-*α* (c), and IFN-*γ* (d), respectively. ∗∗*P* < 0.01.

Next, we estimated the levels of inflammatory cytokines in the serum and brains of the animals. The serum levels of IL-1*β* (Figure 3(a)), IL-6 (Figure 3(b)), TNF-*α* (Figure 3(c)), and IFN-*γ* (Figure 3(d)) levels were increased in the acute phase of sepsis (one day after the surgery, CLP group) as compared with sham mice, suggesting an enhanced systemic inflammatory reaction. In contrast, cortistatins significantly decreased the levels of inflammatory cytokines. Similarly, in the late phase of sepsis (seven days after the surgery), the levels of serum IFN-*γ*, TNF*α*, and IL-1*β* were upregulated in the CLP group compared to the sham group, while the levels of these cytokines were downregulated with cortistatin treatment. Interestingly, the level of IL-6 in the serum returned to normal seven days after the surgery, and cortistatin showed no impact on IL-6 levels at this time point (Figure 3(b)). Similar trends were observed in the cortex (Figure 3(b)).

**Figure 4:**
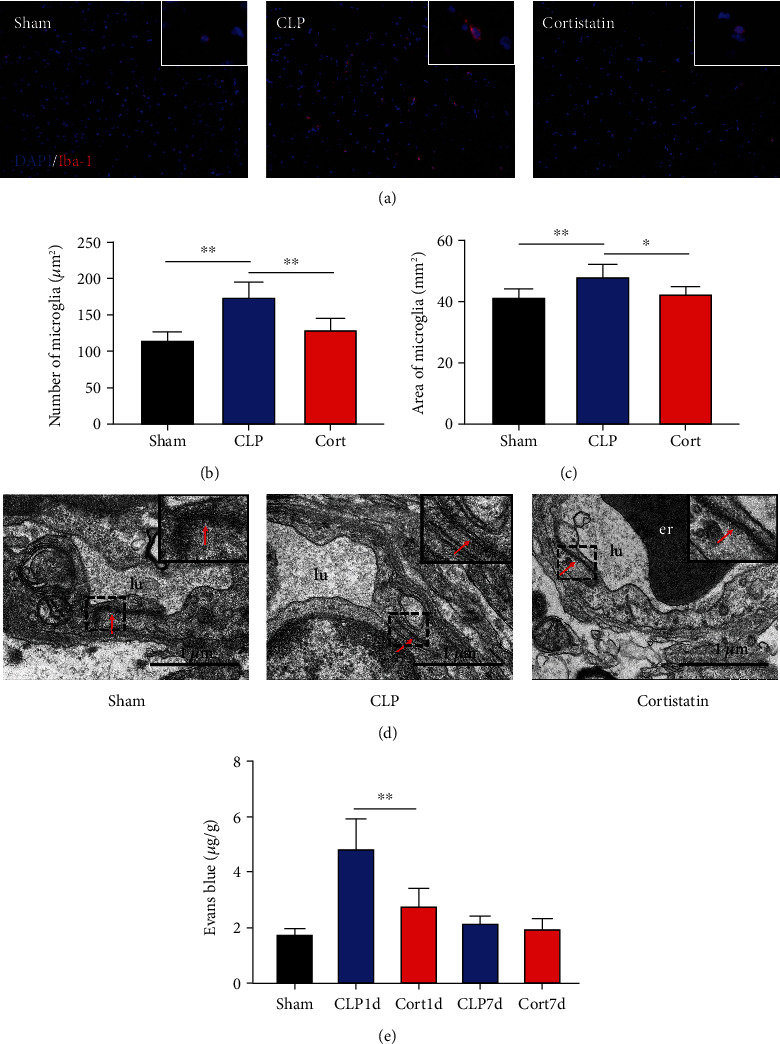
Cortistatin treatment alleviated microglial activation and BBB disruption. (a) Representative Iba1 and DAPI immunofluorescence staining in the prefrontal cortex. Representative magnified Iba1 immunopositive cells were shown in the upper right corner of each group. (b) and (c) Statistical analysis of Iba1 immunopostitive cells counts in the prefrontal cortex. (d) Representative transmission electron micrographs of compact junctions between endothelial cells (red arrows). Representative tight junctions were shown in the upper right corner of each group. (e) Evans blue staining results to investigate the BBB permeability. ∗*P* < 0.05, ∗∗*P* < 0.01. Abbreviations: CLP, cecal ligation and puncture; Cort, cortistatin; er: erythrocyte; lu: lumen.

Microglia are the major local macrophages in the CNS that orchestrate the neuroinflammation. Therefore, we investigated the activation of microglia in the brain. Immunofluorescence was used to evaluate the morphology and distribution of microglia, as investigated by Iba1 immunoreactivity. At 1 and 7 days after surgery, the number of microglia was increased in the frontal cortex. Moreover, as shown in Figure 4(a), the cell body of microglia in CLP mice was enlarged with a thick, shrunken process and aligned with the ameboid morphology of activated microglia. In contrast, microglia had small cell bodies with fine and long processes in the sham and cortistatin groups in the mouse brain, consistent with the amplified morphology of resting microglia. In septic mice, the number of active microglia in the brain was higher than that in the sham group. Treatment with cortistatin significantly decreased the number of microglia (Figure 4(b)). Compared to the CLP group, the area of the cell body was decreased in the cortistatin group (Figure 4(c)).

### 3.4. Cortistatin ameliorated sepsis-induced BBB disruption in the brain of the CLP -induced mice

To explain the neuroprotective effect of cortistatin on the cognition and memory in septic mice, we explored the ultrastructure of the brain using transmission electron microscopy. One day after the CLP surgery, BBB integrity was investigated using transmission electron microscopy (Figure 4(d)). In the sham group, the morphology of the endothelial cells remained normal with no deformation or swelling, the tight junctions of the plasma membrane of adjacent endothelial cells were intact, and no gaps were observed. In the CLP group, disrupted tight junctions and swollen mitochondria in the endothelial cells were observed, and these injuries were clearly attenuated by cortistatin treatment. Similarly, Evans Blue staining also confirmed that, in the acute phase (1 day after septic onset), BBB integrity was disturbed in the brains of septic mice compared with sham mice, and cortistatin treatment could improve Evans Blue extravasation (Figure 4(e)**)**.

## 4. Discussion

In this study, cortistatin treatment improved overall survival and cognitive and behavioral impairments in the CLP-induced sepsis mouse model. Administration of cortistatin alleviated BBB disruption, as revealed by Evans Blue staining. In line with this finding, the BBB ultrastructure showed that disrupted tight junctions and swollen mitochondria in endothelial cells were attenuated by cortistatin treatment. Additionally, we demonstrated that this neuroprotective effect of cortistatin was likely mediated by the reduction in the inflammatory cytokine release and microglial activation.

Sepsis is a severe systemic infectious condition affecting the cardinal organs and is a leading cause of morbidity and mortality worldwide. Involvement of the CNS can result in SAE, affecting up to 71% of patients diagnosed with sepsis [17]. SAE is an acute cerebral dysfunction that manifests as altered mental status, including alteration of consciousness and cognition [18]. In a rat septic model memory deficiency was observed 48 h after CLP surgery [19]. Another study reported that the behavioral deficits of the CLP rats were more severe than those of sham rats [20]. Consistent with previous reports, we also identified impaired cognitive and memory functions in CLP-induced septic mice during the acute and late phases of sepsis, as measured by NORT. In addition, the OFT, TST, and EPMT indicated that sepsis disrupted locomotor activity and exacerbated anxiety-like behaviors in mice. Taken together, these results confirm impaired cognitive and behavioral performance after the onset of sepsis in a mouse model.

The severity of SAE is associated with increased mortality, and one-sixth of patients with sepsis have long-term memory dysfunction, anxiety, depression, and post-traumatic stress disorder [1, 21]. The presence of SAE is critical, and the damage generated by SAE on the CNS appears to be persistent, as shown in an autopsy study [22]. This conclusion at least partly explains the increased mortality and long-term psychocognitive impairment associated with SAE [23]. Nevertheless, the pathophysiology of SAE remains unclear, and there is no available effective treatment for SAE.

The blood-brain barrier (BBB) is a highly selective and dynamic interface between the brain parenchyma and cerebral circulation formed by brain endothelial cells, pericytes, astrocytes, and surrounding microglia [2]. The BBB exerts a critical protective effect against pathogens and toxic threats to the CNS. BBB disruption results in significant alterations in consciousness and cognitive dysfunction [24]. In animal models, lipopolysaccharide (LPS)-induced endotoxemia causes pericyte loss and microvascular dysfunction [25]. In particular, sepsis triggers an excessive immune response, which results in the amplified production of inflammatory cytokines that exert deleterious effects on the integrity and normal function of the BBB [26]. We found that the onset of sepsis resulted in BBB disruption, which was attenuated by cortistatin treatment, as revealed by Evans blue staining. Consistently, observation of the microvasculature ultrastructure in the frontal cortex revealed disrupted tight junctions and swollen mitochondria in the endothelial cells, and these injuries were greatly attenuated by cortistatin treatment. These results prove that systemic inflammatory conditions disrupt BBB integrity and microenvironment homeostasis, which could be attenuated by cortistatin.

Microglial cells are resident phagocytes in the CNS and mediate metabolic and phenotypic changes during sepsis [27]. In various types of CNS diseases, microglia play a harmful role in neuronal loss, BBB leakage, and disease progression following systemic inflammation [4, 28]. Inhibition of microglial activation alleviates BBB permeability and reduces the extent of SAE, although the underlying mechanisms remain poorly understood [2]. Recent reports suggest that microglia migrate to cerebral vessels during sepsis and their activation represents one of the earliest changes observed in SAE [4]. Intravenous administration of LPS also results in microglial activation in the human brain [28]. Similarly, we observed that CLP-induced sepsis resulted in microglial activation in the brain cortex, which was attenuated by cortistatin administration. This effect might explain the alleviation of sepsis-induced BBB disruption.

Disruption of the BBB permits the entry of serum proinflammatory cytokines into the brain [29]. Microglial cells are activated as these proinflammatory cytokines migrated into the brain tissue [30]. Subsequently, an inflammatory signaling cascade is initiated, and several inflammatory mediators are augmented [5, 8]. TNF-*α* is a cardinal proinflammatory cytokine involved in a variety of detrimental effects, including enhanced procoagulant activity of vascular endothelial cells, activation of neurophils and macrophages, and increase in combination with IFN-*γ* in the expression of adherent molecules, resulting in increased microglial adherence to endothelial cells and tissue infiltration [8]. In addition, anti-TNF-*α* antibody treatment downregulates the levels of IL-6 and IFN-*γ* in endotoxemic animals [31], suggesting that TNF-*α* serves as an initial mediator of IL-6 and IFN-*γ*. IFN-*γ* plays a toxic role in LPS-induced shock and polymicrobial sepsis [32]. Administration of recombinant IFN-*γ* prior to sepsis in a rat model significantly reduced T cell apoptosis and natural killer cell activation [33]. Despite the important role of the cytokine storm during the early stage of sepsis, large-scale clinical trials that target cytokines during sepsis have not shown benefits [34]. Consequently, novel alternative therapeutic approaches are urgently required.

Cortistatin is a neuropeptide that was originally found in the cerebral cortex and hypothalamus [35]. Recent studies have revealed that cortistatin is widely expressed in multiple systems and organs, such as the CNS and immune and endocrine systems [35]. Initially, cortistatin was found to be capable of depressing neuronal electricity [36]. In recent years, increasing evidence has strongly suggested a role for cortistatin in inflammation and immunomodulation. In CNS infections, cortistatin has shown therapeutic effects in meningoencephalitis caused by *Klebsiella pneumoniae* in a preclinical study [11]. Cortistatin also alleviates septic shock-associated histopathology, such as the infiltration of inflammatory cells and intravascular disseminated coagulation in the intestine, liver, and lungs [7]. They also found that treatment with cortistatin reduced the local and systemic inflammatory responses in endotoxemic mice [7]. In this study, we also provided evidence that cortistatin exerts a protective effect on overall survival and cognitive and behavioral performance in a septic mouse model.

We also confirmed that cortistatin inhibits the release of systemic and regional inflammatory cytokines during sepsis. This may explain the protective effect of cortistatins on the BBB. Similar to our results, cortistatin prevented the LPS-induced production of cytokines in peritoneal macrophages *in vitro* [37]. In addition, cortistatin treatment leads to a decrease in *K. pneumoniae*-induced proinflammatory cytokine production both *in vivo* in rats and *in vitro* in neuro-glia cocultures, indicating that direct downregulation of glial activity may partially account for the anti-inflammatory effects of cortistatin [10].

However, our study had some limitations. First, our results were obtained using a mouse model, which might be different when applied in a clinical setting. Second, this is a descriptive *in vivo* study demonstrating the therapeutic effect of cortistatin in cognitive defects, BBB disruption, and microglial activation. Thus, further *in vitro* studies are required to explore the potential mechanisms underlying the neuroprotective effects of cortistatin.

## 5. Conclusion

In summary, cortistatin treatment profoundly improved the survival rate and cognitive and emotional dysfunction in a CLP-induced mouse model of sepsis. In addition, cortistatin significantly inhibits BBB disruption, inflammatory cytokine release, and microglial activation. Cortistatin is a potential therapeutic reagent to prevent the frequent sequelae of SAE, including cognitive impairement and functional dependence, and improve the health and quality of life of patients with SAE.

## Data Availability

The data used to support the findings of this study are available from the corresponding authors upon request.

## Supplementary Materials

Supplementary Materials Figure S1 Kaplan-meier survival analysis revealed that cortistatin improved the overall survival of the CLP mice in a dose-dependent manner.
