# Supplementary material for: Cortistatin-14 Exerts Neuroprotective Effect Against Microglial Activation, Blood-brain Barrier Disruption, and Cognitive Impairment in Sepsis-associated Encephalopathy
Source: J Immunol Res. 2022 Sep 13;2022:3334145. doi: 10.1155/2022/3334145 (PMC9489378; doi:10.1155/2022/3334145)
Supplement: Supplementary Materials — Figure S1 Kaplan-meier survival analysis revealed that cortistatin improved the overall survival of the CLP mice in a dose-dependent manner. [file 3334145.f1.doc]

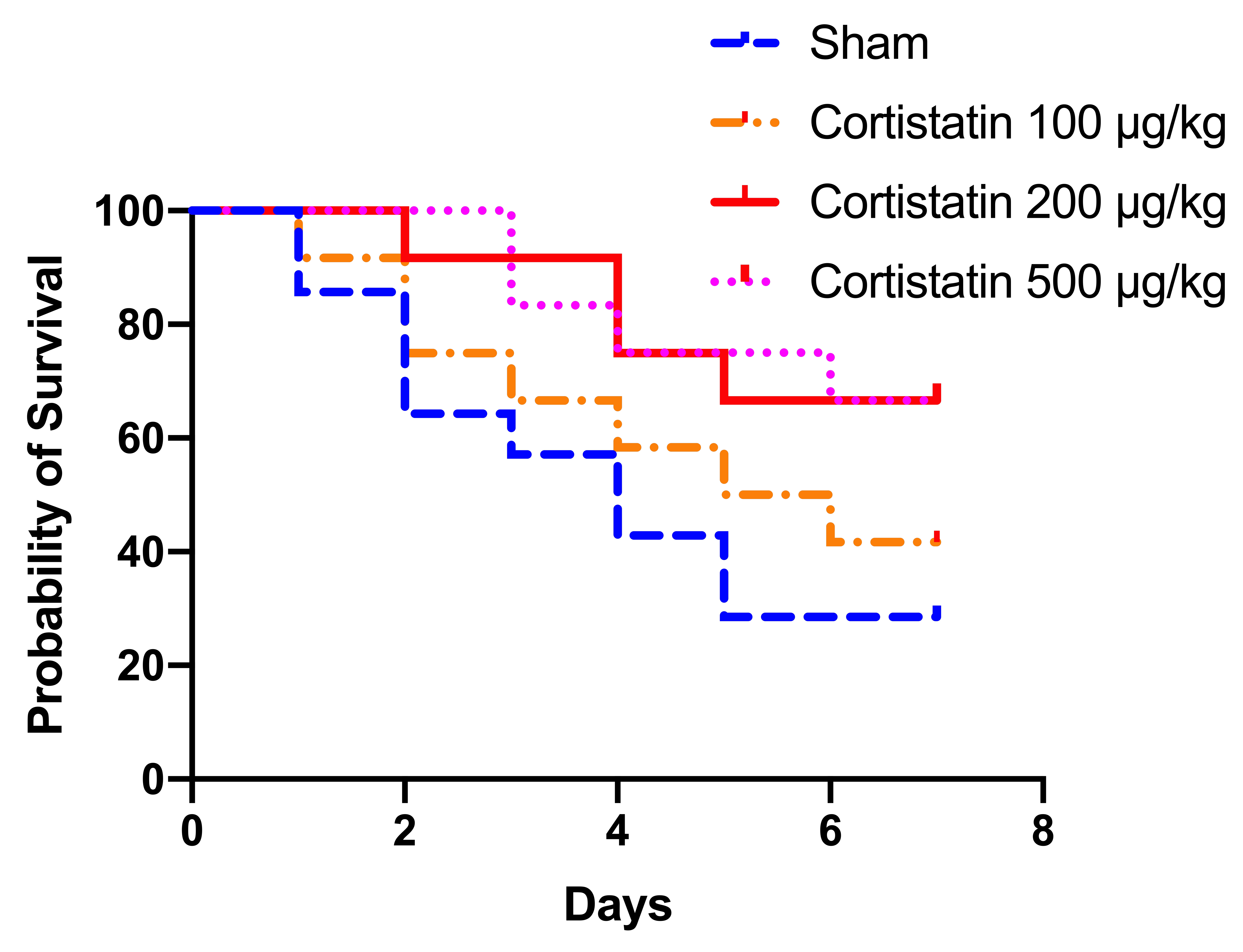


**Figure S1** Kaplan-meier survival analysis revealed that cortistatin improved the overall survival of the CLP mice in a dose-dependent manner.
